# Association between triglyceride-glucose index and low-density lipoprotein particle size in korean obese adults

**DOI:** 10.1186/s12944-023-01857-5

**Published:** 2023-07-04

**Authors:** Sanghoon Kim, Ji-Won Lee, Yaeji Lee, Youhyun Song, John A Linton

**Affiliations:** 1 Department of Family Medicine, Severance Hospital, Yonsei University College of Medicine, Seoul, 03722 Republic of Korea; 2 Institute for Innovation in Digital Healthcare, Yonsei University, Seoul, 06237 Republic of Korea; 3 Department of Biostatistics and Computing, Yonsei University, Seoul, 03722 Republic of Korea; 4 Healthcare Research Team, Health Promotion Center, Gangnam Severance Hospital, Yonsei University College of Medicine, Seoul, 06273 Republic of Korea; 5 International Health Care Center, Severance Hospital, Yonsei University Health System, Seoul, 03722 Republic of Korea

**Keywords:** Triglyceride-glucose index, Low-density lipoprotein subfractions, Small dense low-density lipoprotein cholesterol, Cardiovascular disease, Insulin resistance, Atherosclerosis, Atherogenic lipoprotein parameter, Diabetic dyslipidemia

## Abstract

**Background:**

Small dense low-density lipoprotein cholesterol (sdLDL-C) is the lipoprotein marker among the various lipoproteins that is most strongly related to atherosclerosis. Insulin resistance (IR) can alter lipid metabolism, and sdLDL-C is characteristic of diabetic dyslipidemia. Therefore, this study sought to inspect the relationship between the triglyceride-glucose (TyG) index and mean low-density lipoprotein (LDL) particle size.

**Methods:**

In this study, a total of 128 adults participated. The correlation coefficients between various lipoproteins and the TyG index were compared using Steiger’s Z test and the Spearman correlation. The independent link between the TyG index and mean LDL particle size was demonstrated by multiple linear regression analysis. To identify the TyG index cutoff value for the predominance of sdLDL particles, receiver operating characteristic curves were plotted.

**Results:**

Mean LDL particle size correlated more strongly with the TyG index than did very low-density lipoprotein, low-density lipoprotein cholesterol, and high-density lipoprotein cholesterol. Regression analysis demonstrated that mean LDL particle size had a strong association with the TyG index (β coefficient = -0.038, *P*-value < 0.001). The TyG index optimal cutoff value for sdLDL particle predominance and the corresponding area under the curve (standard error: 0.028, 95% confidence interval: 0.842–0.952) were 8.72 and 0.897, respectively, which were close to the cutoff value of diabetes risk in Koreans.

**Conclusions:**

Mean LDL particle size is more strongly correlated with the TyG index than do other lipid parameters. After correcting for confounding variables, mean LDL particle size is independently linked with the TyG index. The study indicates that the TyG index is strongly related to atherogenic sdLDL particles predominance.

**Supplementary Information:**

The online version contains supplementary material available at 10.1186/s12944-023-01857-5.

## Background

In 2019, 17.9 million people died due to cardiovascular disease (CVD), which was the major cause of death in 32% of all fatalities worldwide [1, 2]. According to the Korean Statistical Information Service, CVD is South Korea’s second major cause of death [3]. CVD has become more prevalent in recent decades with the rapidly aging population in South Korea, resulting in higher mortality and hospitalization rates [4]. Hence, CVD is a financial burden for patients and a public health issue nationally and internationally [2, 4].

Low-density lipoprotein cholesterol (LDL-C) contributes to an escalated CVD risk [5]. However, small dense LDL cholesterol (sdLDL-C), an emerging CVD risk biomarker and an independent CVD risk factor, is a stronger CVD predictor than traditional LDL-C and was discovered to be independent of other major risk factors [6–8]. A smaller mean LDL particle size is associated with metabolic syndrome and a greater CVD risk, sometimes even when LDL-C levels are normal [8, 9]. Rather than larger LDL particles, smaller LDL particles are more likely to infiltrate the wall of artery and adhere to proteoglycans [10]. Moreover, sdLDL-C is easily oxidized and secretes pro-inflammatory cytokines, which promote atherosclerosis, thereby raising ischemic heart disease risk [11, 12]. In the recent prospective study, researchers discovered that sdLDL-C has the most atherogenic potential among other atherogenic lipoproteins [13].

Insulin resistance (IR) is defined as reduced sensitivity and reaction to insulin secretion [14]. IR increases CVD risk independently of other cardiovascular risk factors [15]. CVD is frequent in individuals with metabolic syndrome, obesity, high IR, and type 2 diabetes mellitus [16, 17]. Patients with IR likely have an increased prevalence of sdLDL particles [18]. Additionally, various components of the IR syndrome are related to sdLDL-C, including hyperinsulinemia, low serum high-density lipoprotein cholesterol (HDL-C) levels, hypertension, diabetes, and hypertriglyceridemia [19].

The glucose clamp technique is considered to be the gold standard for assessing insulin secretion and resistance; however, it is labor-intensive and invasive. Therefore, simple alternatives to the clamp technique have been extensively validated [20]. Logarithmizing the product of fasting plasma glucose level and serum triglyceride concentration yields the triglyceride-glucose (TyG) index, and this alternative biomarker is one of the most promising ones [21]. In identifying IR, when compared to the homeostatic model assessment (HOMA)-IR index, the TyG index showed superiority [21, 22]. Moreover, the TyG index predicts various chronic diseases by reflecting IR and systemic inflammation [23] and is linked to CVD progress and prognosis [22].

Earlier researches have indicated an association between sdLDL-C and IR. However, data on this association are limited. Moreover, several studies on the relationship between sdLDL particles and IR have been contradictory, with the relationship markedly dependent on serum triglyceride concentration [19].

Therefore, this research sought to examine the relationship between the TyG index, a biomarker of IR and mean LDL particle size, which decreases as sdLDL-C increases. This is the first investigation into the relationship.

## Methods

### Aim of the study

The investigation’s goal was to determine how the TyG index and mean LDL particle size are related.

### Study population and design

During October 2016 and September 2021, this study involved 272 participants who visited the Severance Hospital’s Family Medicine Department for health checkups. The inclusion criteria were patients who had no history of malignancy or thyroid, renal, or cardiovascular disease.

**Fig. 1:**
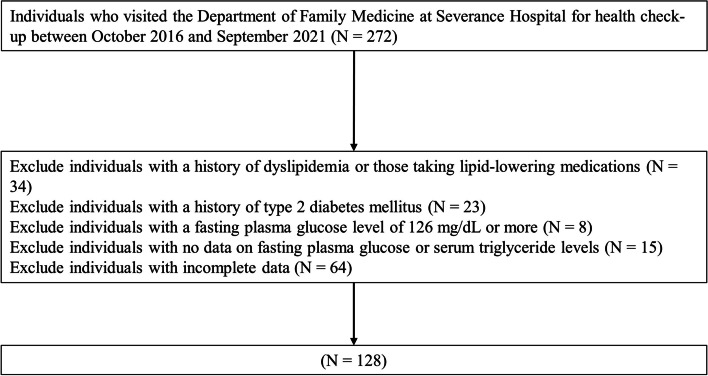
Flow chart for selecting the research population

The exclusion criteria were patients with (1) history of cholesterol-lowering drugs or diagnosis of dyslipidemia; (2) a history of type 2 diabetes mellitus or fasting plasma glucose level of 126 mg/dL or higher; (3) no data on fasting plasma glucose or serum triglyceride level; (4) incomplete data. Finally, the analysis included 128 eligible participants (Fig. 1). Personally identifiable information was anonymized, and patient risks were minimized using a retrograde cross-sectional design. The Severance Hospital’s Institutional Review Board approved the study, which followed the guidelines of the Declaration of Helsinki (Approval number: 4-2022-1103).

### Clinical and body composition measurement

The medical and social history information from patients was obtained through self-administered questionnaires. Self-reported smoking and weekly alcohol consumption of more than 72 g were defined as “smoking” and “alcohol consumption,“ respectively.

An electronic manometer (BPBio 320; Biospace, Seoul, South Korea) measured systolic and diastolic blood pressure from the right arm, with the sitting participant. A heart rate monitor (Polar-FS3c; Polar Electro Oy, Kempele, Finland) was used to measure the resting heart rate. Using a stadiometer, height was obtained to the closest 0.1 cm. With a bioelectrical impedance analyzer (Inbody 720; Biospace, Seoul, South Korea), body weight and body composition were evaluated. By dividing each individual’s weight by the square of their height, the body mass index (BMI) for each individual was obtained. In a standing position, waist circumference was gauged at the center between the lowest rib and the iliac crest. One trained professional obtained all measurements. Using computed tomography (CT), the visceral and subcutaneous abdominal fat areas were calculated (Tomoscan 350; Philips, Mahwah, New Jersey, USA) [24].

### Laboratory measurements

All blood tests were performed on patients who had fasted for 12 h the night before. Fasting plasma glucose, uric acid, total cholesterol, aspartate transaminase (AST), alanine transaminase (ALT), gamma-glutamyl transferase (GGT), high-sensitivity C-reactive protein (hsCRP), HDL-C, and LDL-C levels were examined via an automatic chemical analyzer (Hitachi 7600; High-Technologies Corporation, Hitachi, Tokyo, Japan). Fasting insulin level was obtained by an immunology analyzer (Elecsys 2010; Roche, Indianapolis, Indiana, USA). Following previous procedures [25, 26], this study used the Quantimetrix Lipoprint™ system with polyacrylamide tube gel electrophoresis to determine mean LDL particle size and subfractions. Based on the retention factor (Rf, that is, relative mobility) between the HDL-C fraction (Rf = 1.0) and the very low-density lipoprotein cholesterol (VLDL-C) fraction (Rf = 0.0), the Rfs of the LDL subclasses from 1 to 7 were estimated to be 0.32, 0.38, 0.45, 0.51, 0.56, 0.60, and 0.64, respectively. The large, buoyant LDL (lbLDL) subfractions were defined as the LDL subclasses 1–2, whereas the sdLDL subfractions corresponded to the LDL subclasses 3–7 [27]. Furthermore, the system computed the mean LDL particle size. And it was divided into three categories according to the size. LDL particles with a mean size equal to or greater than 268.0 Angstrom (Å) were categorized as the LDL subclass pattern A, while a mean size below 265.0 Å or between 265.0 Å and 268.0 Å corresponded to the LDL subclass patterns B or I, respectively.

The TyG index was obtained through the following equation: ln [Triglyceride (mg/dl) × glucose (mg/dl)/2] and divided into quartiles. The quartile ranges were as follows: TyG index group 1 [7.56–8.35]; group 2 [8.35–8.63]; group 3 [8.64–9.03]; group 4 [9.03–10.24]. The following formula was used to determine the HOMA-IR: [Fasting serum insulin (µU/mL) × Fasting plasma glucose (mg/dL) divided by 405] [28]. The Framingham risk score (FRS) was calculated using age, total cholesterol level, smoking history, HDL-C level, and systolic blood pressure to assess cardiovascular risk [29].

### Sample size calculation

Although the prevalence of the LDL subclass pattern B, or sdLDL predominance, in obese adults, has not been precisely investigated, it has been estimated to be 17–50% in some studies [30–32]. Conservatively assuming a prevalence of 15%, the sample size for estimating the area under the curve (AUC) using power analysis with a 95% confidence level and a width of confidence interval of 0.196 was 113 [33].

### Statistical analysis

Categorical variables are expressed as frequencies and percentages. For properly distributed data, continuous variables are presented as means and standard deviations. Non-normally distributed data are reported as medians and ranges. Using the Kruskal–Wallis test for non-normally distributed variables and the analysis of variance for normally distributed variables, the baseline features of the TyG index quartile groups were compared. In contrast, proportions were compared using Pearson’s chi-square test. The TyG index and clinical variables were correlated using the Spearman correlation method. When analyzing the dependency of two correlation coefficients, Steiger’s Z tests were applied for identifying the differences in absolute correlation coefficients with lipid profiles and the TyG index. To analyze the risk factors of the TyG index, multivariable linear regression models were built, with confounding variables selected based on clinical knowledge. The linear regression results are expressed as beta coefficients, confidence intervals (CIs), and *P*-values. Linear trends in the percentages of the 10-year cardiovascular risk among the TyG index quartile groups were investigated using the Cochran–Armitage trend test for linear trend. To calculate the AUC and establish the cutoff value for the TyG index of sdLDL particles dominance, receiver operating characteristic (ROC) curves were created. DeLong’s method was used to obtain the standard error (SE) and 95% CI of the AUC. Two-sided *P*-values of less than 0.05 were considered statistically significant. The statistical analyses were carried out by using R, version 4.2.2 (R Foundation for Statistical Computing, Vienna, Austria, http://www.R-project.org).

## Results

### Clinical characteristics of the participants

The clinical features of the participants are shown in Table 1 based on the TyG index quartiles. Men represented 22.7% of the 128 participants included in the study. The average BMI and age of study participants were 30.6 and 44.9 years, respectively. Participants in the highest TyG quartile showed a greater waist circumference (*P* = 0.031) and abdominal visceral fat area (*P* = 0.043). However, no correlation with the abdominal subcutaneous fat area was shown (*P* = 0.657). Additionally, participants in the highest TyG quartile had increased serum AST (*P* = 0.006), ALT (*P* = 0.003), GGT (*P* < 0.001), uric acid (*P* < 0.001), and insulin levels (*P* < 0.001), as well as elevated HOMA-IR (*P* < 0.001) and FRS (*P* = 0.011).

**Table 1:** Clinical features of all participants and features according to TyG index quartiles

Variable	Total (*n* = 128)	Quartile 1 [7.86– 8.39] (*n* = 32)	Quartile 2 (8.41– 8.71) (*n* = 32)	Quartile 3 (8.71– 9.08) (*n* = 32)	Quartile 4 (9.10– 9.99) (*n* = 32)	*P*-value
Age (years)	44.86 ± 10.07	44.28 ± 10.85	45.64 ± 10.54	46.16 ± 9.89	43.34 ± 9.13	0.674
Sex						0.021
Male	29.0 (22.66%)	2.0 (6.25%)	6.0 (18.75%)	9.0 (28.12%)	12.0 (37.50%)	
Female	99.0 (77.34%)	30.0 (93.75%)	26.0 (81.25%)	23.0 (71.88%)	20.0 (62.50%)	
Body mass index (kg/m²)	30.63 ± 4.95	29.20 ± 4.90	30.81 ± 5.38	31.30 ± 4.70	31.27 ± 4.76	0.300
Waist circumference (cm)	97.44 ± 12.19	92.82 ± 11.14	96.73 ± 14.02	98.21 ± 9.97	102.01 ± 11.94	0.031
Systolic blood pressure (mmHg)	124.24 ± 14.34	121.88 ± 13.34	123.35 ± 15.03	126.19 ± 12.85	125.42 ± 16.20	0.64
Diastolic blood pressure (mmHg)	74.74 ± 10.12	72.80 ± 9.03	74.28 ± 9.49	75.68 ± 8.16	76.13 ± 13.17	0.574
Heart rate (beats/minute)	71.80 ± 10.65	70.87 ± 9.41	74.67 ± 10.96	68.68 ± 11.22	73.03 ± 10.46	0.139
AST (IU/L)	24.00 (11.00–93.00)	22.00 (14.00–46.00)	22.00 (14.00–81.00)	24.50 (15.00–74.00)	27.50 (11.00–93.00)	0.006
ALT (IU/L)	25.00 (4.00–280.00)	18.00 (8.00–64.00)	24.50 (11.00–178.00)	27.00 (4.00–280.00)	30.50 (6.00–231.00)	0.003
GGT (IU/L)	21.50 (7.00–155.00)	12.50 (7.00–30.00)	29.00 (9.00–155.00)	21.50 (10.00–89.00)	26.00 (10.00–141.00)	< 0.001
hsCRP (mg/L)	1.20 (0.10–16.00)	1.00 (0.10–16.00)	1.20 (0.10–15.30)	1.10 (0.20–11.60)	1.20 (0.20–7.70)	0.945
Uric acid (mg/dL)	5.49 ± 1.17	4.99 ± 1.09	5.24 ± 0.93	5.69 ± 1.03	6.06 ± 1.32	< 0.001
Insulin (µU/mL)	10.80 (1.10–45.50)	6.70 (1.10–40.60)	11.00 (3.80–31.60)	9.85 (2.90–30.40)	18.25 (4.50–45.50)	< 0.001
HOMA-IR	49.29 (4.20–223.44)	30.51 (4.20–209.32)	48.11 (14.52–133.42)	47.26 (11.21–137.81)	81.44 (20.40–223.44)	< 0.001
Triglyceride	148.32 ± 83.04	75.84 ± 12.42	104.31 ± 12.37	143.66 ± 17.63	269.47 ± 70.96	< 0.001
Glucose	98.84 ± 9.07	95.78 ± 7.67	98.59 ± 9.10	99.19 ± 8.54	101.81 ± 10.16	0.065
TyG Index	8.77 ± 0.51	8.18 ± 0.17	8.53 ± 0.09	8.86 ± 0.11	9.49 ± 0.26	< 0.001
Abdominal visceral fat area (cm^2^)	64.30 (25.70–137.00)	56.30 (25.70–105.00)	60.50 (33.40–128.00)	66.70 (40.00–106.00)	69.70 (41.60–137.00)	0.043
Abdominal subcutaneous fat area (cm^2^)	109.00 (42.60–311.00)	115.00 (42.60–297.00)	95.90 (57.20–201.00)	108.00 (52.80–248.00)	125.50 (43.90–311.00)	0.657
Smoking history						0.004
Yes	12.0 (9.38%)	2.0 (6.25%)	0.0 (0.00%)	2.0 (6.25%)	8.0 (25.00%)	
No	116.0 (90.62%)	30.0 (93.75%)	32.0 (100.00%)	30.0 (93.75%)	24.0 (75.00%)	
Alcohol consumption						0.200
Yes	27.0 (21.09%)	7.0 (21.88%)	3.0 (9.38%)	7.0 (21.88%)	10.0 (31.25%)	
No	101.0 (78.91%)	25.0 (78.12%)	29.0 (90.62%)	25.0 (78.12%)	22.0 (68.75%)	
Hypertension						0.924
Yes	24.0 (33.33%)	6.0 (28.57%)	5.0 (31.25%)	6.0 (37.50%)	7.0 (36.84%)	
No	48.0 (66.67%)	15.0 (71.43%)	11.0 (68.75%)	10.0 (62.50%)	12.0 (63.16%)	
Framingham risk score	3.53 (1.00–18.55)	2.41 (1.00–10.04)	3.09 (1.03–13.05)	4.02 (1.25–13.58)	5.16 (1.40–18.55)	0.011

Values are presented as means ± standard deviations, medians (ranges) or numbers (%)

*Abbreviations*: *AST *Aspartate aminotransferase, *ALT *Alanine aminotransferase, *GGT *Gamma- glutamyl transferase, *hsCRP *High-sensitivity C-reactive protein, *HOMA-IR *Homeostasis model assessment-estimated insulin resistance

Table 2 presents the lipid and LDL subfractions profiles based on the TyG index quartiles. Higher serum total cholesterol (*P* = 0.003), VLDL-C (*P* < 0.001), LDL-C (*P* < 0.001), and sdLDL-C (*P* < 0.001) levels as well as a greater proportion of sdLDL-C and sdLDL:lbLDL ratio (*P* < 0.001) were found in participants with the highest TyG index values. Conversely, these participants showed lower HDL-C (*P* < 0.001) and lbLDL-C (*P* < 0.001) levels and a lower mean LDL particle size (*P* < 0.001).

**Table 2:** Lipid and LDL subfractions profiles based on the TyG index quartiles

Variable	Total (*n *= 128)	Quartile 1 [7.86– 8.39] (*n* = 32)	Quartile 2 (8.41– 8.71) (*n* = 32)	Quartile 3 (8.71– 9.08) (*n* = 32)	Quartile 4 (9.10– 9.99) (*n* = 32)	*P*-value
Total cholesterol (mg/dL)	198.00 (116.00–335.00)	186.00 (116.00–295.00)	190.00 (147.00–279.00)	198.00 (130.00–282.00)	217.50 (156.00–335.00)	0.003
HDL-C (mg/dL)	53.50 (33.00–94.00)	59.50 (38.00–91.00)	55.00 (36.00–94.00)	50.00 (34.00–75.00)	45.50 (33.00–68.00)	< 0.001
LDL-C (mg/dL)	124.50 (68.00–205.00)	113.00 (70.00–205.00)	117.00 (75.00–186.00)	128.00 (68.00–186.00)	136.50 (73.00–200.00)	0.001
VLDL-C (mg/dL)	19.53 ± 4.67	16.33 ± 3.14	18.10 ± 4.04	20.02 ± 3.24	23.65 ± 4.70	< 0.001
lbLDL-C (mg/dL)	31.16 (0.71, 54.45)	33.13 (15.32, 51.99)	36.30 (14.30, 54.45)	31.70 (0.71, 46.62)	23.69 (8.46, 42.78)	< 0.001
sdLDL-C (mg/dL)	3.26 (0.00, 26.18)	0.85 (0.00, 8.70)	2.12 (0.00, 18.06)	4.63 (0.00, 21.48)	14.21 (4.55, 26.18)	< 0.001
Percent sdLDL-C (%)	8.62 (0.00–60.00)	2.10 (0.00–18.93)	5.98 (0.00–55.80)	12.65 (0.00–44.08)	38.28 (14.60–60.00)	< 0.001
sdLDL-C:lbLDL-C ratio	0.09 (0.00–1.50)	0.02 (0.00–0.23)	0.06 (0.00–1.26)	0.14 (0.00–0.79)	0.62 (0.17–1.50)	< 0.001
Mean LDL particle size (Å)	267.55 (244.60–274.80)	272.10 (262.80–274.80)	269.05 (250.20–274.80)	265.80 (255.90–272.40)	257.65 (244.60–265.90)	< 0.001

Values are presented as means ± standard deviations, medians (ranges) or numbers (%)

*Abbreviations*: *HDL-C *High-density lipoprotein cholesterol, *LDL-C *Low-density lipoprotein cholesterol, *VLDL-C *Very low-density lipoprotein cholesterol, *lbLDL-C *Large buoyant low-density lipoprotein cholesterol, *sdLDL-C *Small dense low-density lipoprotein cholesterol

### Correlation between TyG index and clinical variables

**Fig. 2:**
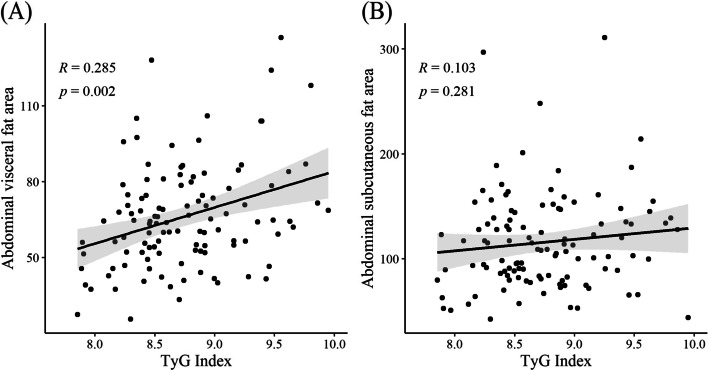
Relationship between abdominal visceral and abdominal subcutaneous fat areas on CT and the TyG index. **a** TyG index and abdominal visceral fat area and **b** TyG index and abdominal subcutaneous fat area. CT, computed tomography; TyG, triglyceride-glucose

AST (R = 0.33, *P* < 0.001), ALT (R = 0.335, *P* < 0.001), GGT (R = 0.428, *P* < 0.001), uric acid (R = 0.36, *P* < 0.001), and insulin (R = 0.401, *P* < 0.001) levels as well as HOMA-IR (R = 0.412, *P* < 0.001) and FRS (R = 0.315, *P* < 0.001) all correlated with the TyG index (Additional file 1-1). The abdominal visceral fat area also showed a significant relationship (R = 0.285, *P* = 0.002). Additionally, the TyG index indicated a positive correlation with waist circumference (R = 0.262, *P* = 0.004). However, there was no relationship with BMI (R = 0.153, *P =* 0.095). Other insulin markers were also analyzed **(**Additional file 1–2**)**. Especially in relation to FRS, the TyG index was superior to HOMA-IR (R = 0.06, *P =* 0.52) and Triglyceride/HDL-C (R = 0.296, *P <* 0.001). This research indicated a correlation between the distribution of abdominal fat and the TyG index, with the TyG index having a positive correlation with the abdominal visceral fat area (R = 0.285, *P* = 0.002), but no substantial correlation with the abdominal subcutaneous fat area (R = 0.103, *P* = 0.281) **(**Fig. 2**)**. In addition, the abdominal visceral fat area showed a significant connection with mean LDL particle size. However, there was no relationship between the abdominal subcutaneous fat area and the TyG index [34, 35] (Additional file 2).

### Comparisons of correlation coefficients of lipid profiles and the TyG index

**Fig. 3:**
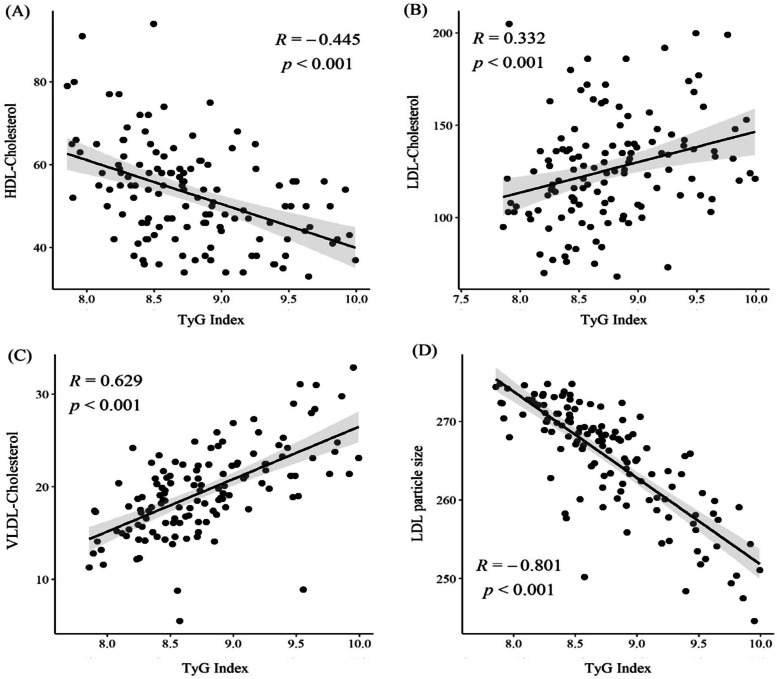
Relationship between lipid profiles and TyG index. **a** TyG index and HDL-C level; **b** TyG index and LDL-C level; **c** TyG index and VLDL level; **d** TyG index and mean LDL-particle size. The *P*-value was calculated using the Spearman correlation coefficient. HDL-C, high-density lipoprotein-cholesterol; LDL-C, low-density lipoprotein cholesterol; TyG, triglyceride-glucose

Figure 3 illustrates the associations between various lipid profiles and the TyG index. The presented lipid profiles were all significantly related to the TyG index. HDL-C (R = -0.445, *P* < 0.001) and mean LDLp (R = -0.801, *P* < 0.001) showed a negative correlation while LDL-C (R = 0.332, *P* < 0.001) and VLDL-C (R = 0.629, *P* < 0.001) showed a positive correlation with the TyG index. The study compared the correlation coefficients between the lipid profiles and the TyG index by applying Steiger’s Z test and a model with BMI, sex and age adjustments (Table 3). Based on a Spearman correlation analysis, the TyG index had a stronger correlation with LDL particle size (*r* = -0.755, *P* ^b^ < 0.001) than with HDL-C levels (*r* = -0.417, *P* ^a^ < 0.001; Steiger’s Z test, *P* ^b^ < 0.001), LDL-C levels (*r* = 0.298, *P* ^a^= 0.001; Steiger’s Z test, *P* ^b^ < 0.001), and VLDL-C levels (*r* = -0.603, *P* ^a^ < 0.001; Steiger’s Z test, *P* ^b^ < 0.001).

**Table 3:** Comparison between correlation coefficients for the TyG index

Variable	r	*P*-value^a^	*P*-value^b^
TyG index			
HDL-C	-0.417	< 0.001	< 0.001
LDL-C	0.298	0.001	< 0.001
VLDL-C	0.603	< 0.001	< 0.001
Mean LDL particle size	-0.755	< 0.001	Ref*

Between the TyG index and HDL-C, LDL-C, and VLDL-C levels and mean LDL particle size, partial correlation coefficients are defined as r values, adjusted for age, sex, and BMI

*Abbreviations*: *HDL-C *High-density lipoprotein cholesterol, *LDL-C *Low-density lipoprotein cholesterol, *VLDL-C *Very low-density lipoprotein cholesterol

* The reference value is defined as R between the TyG index and mean LDL particle size

^a^ *P*-values for r between the TyG index and HDL-C, LDL-C, VLDL-C levels and LDL particle size

^b^ *P*-values for comparing absolute correlation coefficients via Steiger’s Z test between the TyG Index and HDL-C, LDL-C, and VLDL-C levels and mean LDL particle size

### Independent correlation of TyG index with mean LDL particle size and optimal cutoff value for sdLDL particles predominance

Table 4 shows the independent correlation of the TyG index with clinical and metabolic variables. LDL-C (*P* = 0.027), VLDL-C (*P* = 0.000), mean LDL-particle size (*P* = 0.000), and insulin levels (*P* = 0.001) were identified as notable explanatory variables for the TyG index, together accounting for 76.75% of the variance in the TyG index. The explanatory variables were not inferior to other IR markers when compared in multiple linear regression analysis (Additional file 1–3).

**Table 4:** Multiple linear regression analysis to determine relationships between the TyG index and clinical metabolic variables

Variable	βcoefficient	CI	*P*-value
Age (years)	0.001	(-0.004–0.006)	0.726
Sex			
Male	Ref		
Female	0.048	(-0.08–0.177)	0.455
Body mass index (kg/m²)	-0.009	(-0.021–0.003)	0.134
Alcohol consumption			
Yes	0.018	(-0.144–0.107)	0.775
No	Ref		
Smoking history			
Yes	0.082	(-0.26–0.096)	0.361
No	Ref		
Systolic blood pressure (mmHg)	-0.001	(-0.007–0.005)	0.712
Diastolic blood pressure (mmHg)	0.006	(-0.003–0.014)	0.186
LDL-C (mg/dL)	0.002	(0.000–0.004)	0.027
VLDL-C (mg/dL)	0.041	(0.029–0.052)	0.000
Mean LDL particle size (Å)	-0.038	(-0.047– -0.03)	0.000
Uric acid (mg/dL)	0.011	(-0.035–0.057)	0.626
Insulin (µU/mL)	0.010	(0.004–0.016)	0.001

Adjusted R-squared: 0.7675

*Abbreviations*: *CI *Confidence interval, *LDL-C *Low-density lipoprotein cholesterol, *VLDL-C *Very low-density lipoprotein cholesterol

**Fig. 4:**
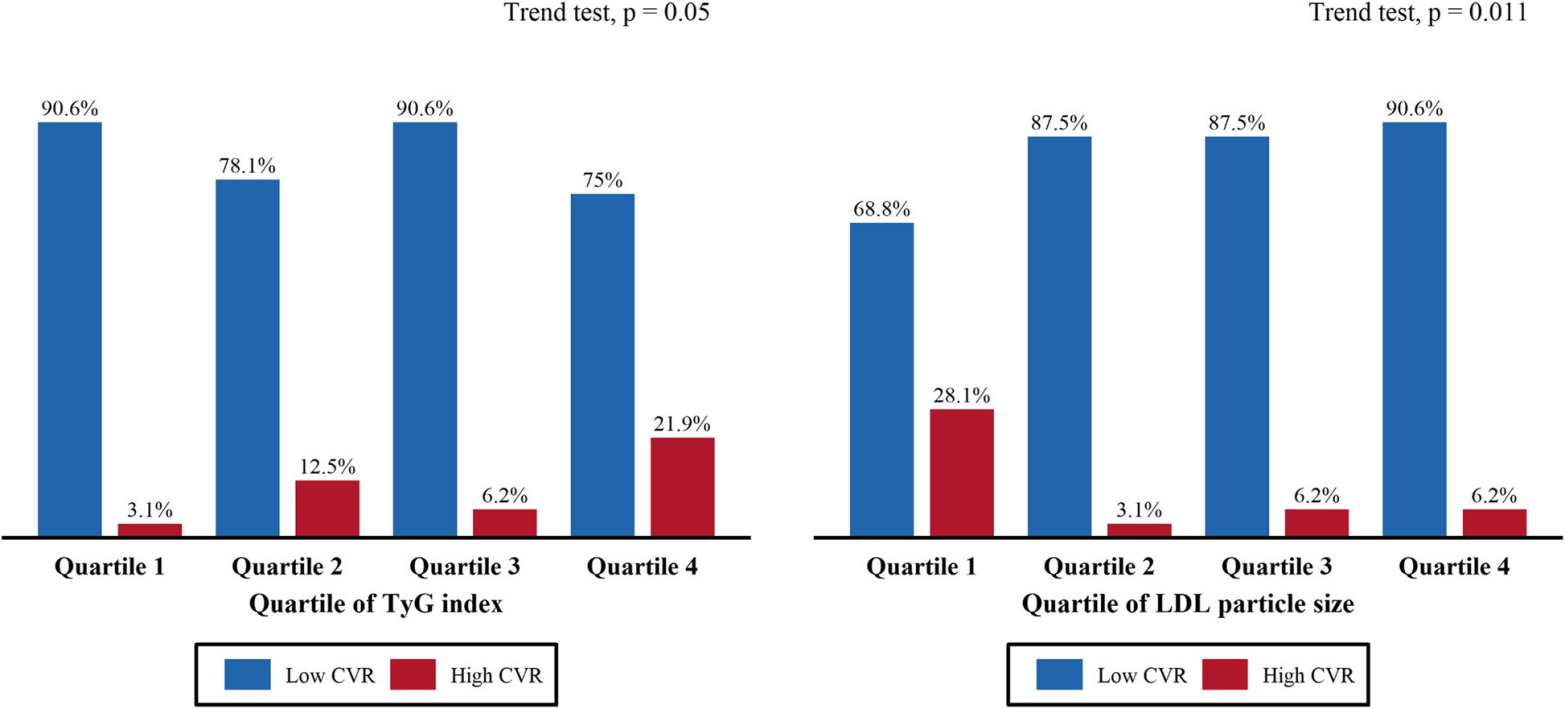
CVR using FRS based on TyG index quartile and LDL particle size quartile. Percentage of 10-year CVR using FRS. Those with a score < 10% were classified as low risk and those with a score > = 10% were classified as high risk. CVR, cardiovascular risk; FRS, Framingham risk score; LDL, low-density lipoprotein; TyG, triglyceride-glucose

This study also used the FRS to assess the percentages of the 10-year cardiovascular risk among the TyG index quartiles and the LDL particle size quartiles. Participants with an FRS of 10% or less were considered at low CVD risk, while those with an FRS of 10% or more were at high CVD risk. The ratio of participants with high CVD risk increased in the higher TyG index quartiles and in the lower LDL particle size quartiles. On the contrary, the ratio of participants with low CVD risk decreased in the lower TyG index quartiles and in the higher LDL particle size quartiles (Fig. 4).

**Fig. 5:**
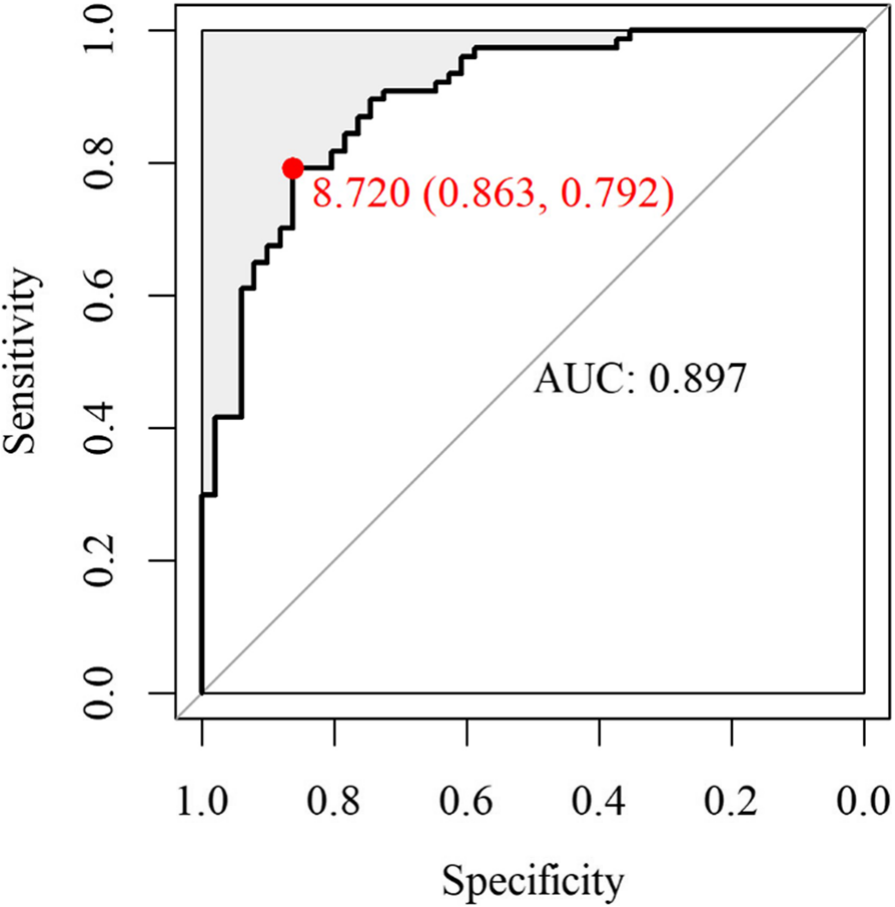
Receiver operating characteristic curve analysis to obtain an appropriate value for the TyG index predictable for small, dense LDL particles (subclass pattern B) predominance. AUC, area under the curve; LDL, low-density lipoprotein; TyG, triglyceride-glucose

The optimal cutoff value for sdLDL particle predominance (subclass pattern B) was determined using ROC curve analysis (Fig. 5). The AUC was 0.897 (SE: 0.028, 95% CI: 0.842–0.952), and the cutoff value was 8.72, which was close to 8.8, the cutoff value for diabetes risk in Koreans [36].

**Fig. 6:**
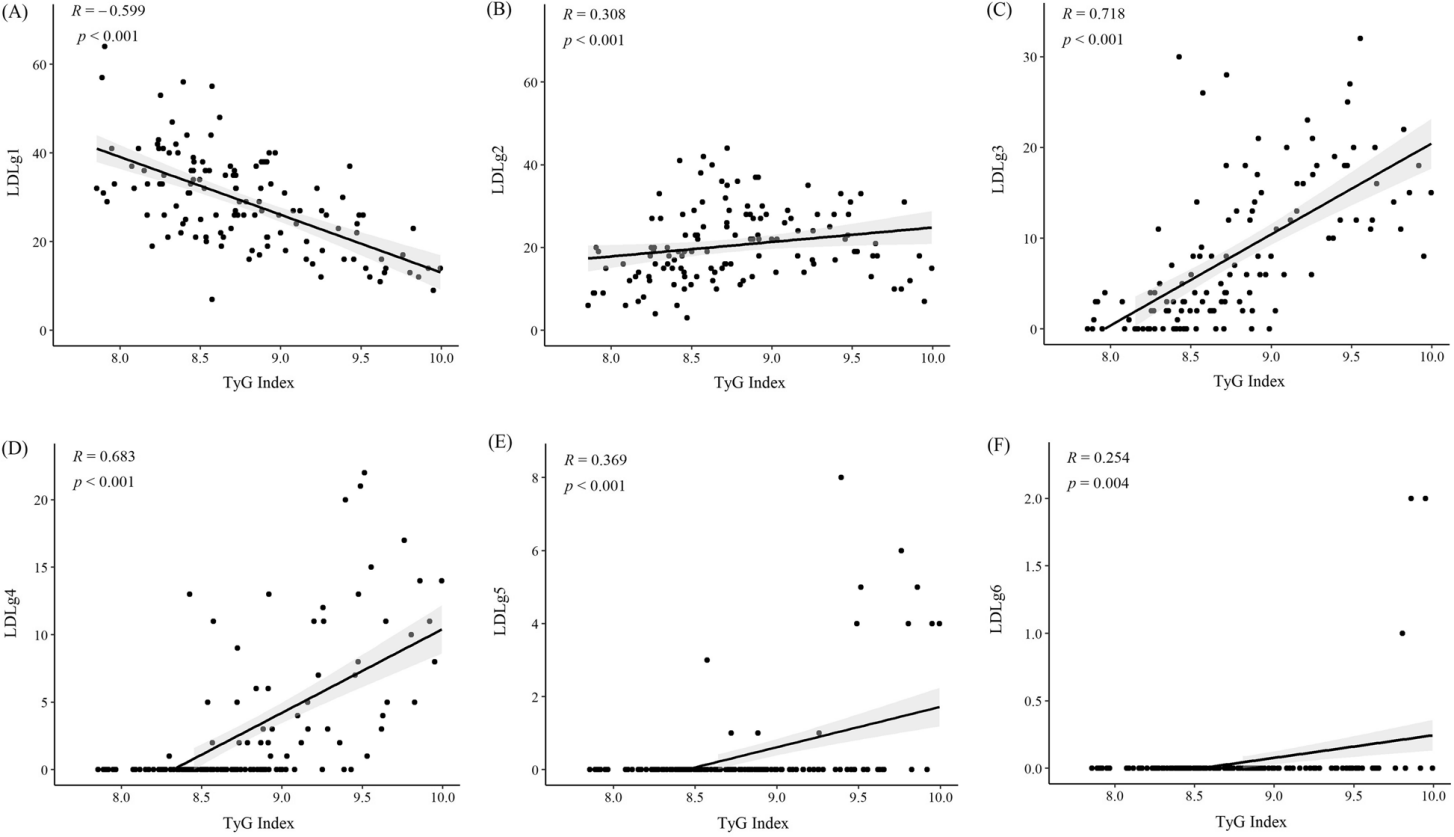
LDL subfractions concentration distribution according to the TyG index. LDLg1 to LDLg6 represent concentrations (mg/dL) of LDL subclasses 1 to 6, respectively. LDL subclass 7 was not listed because it was not found. LDL, low-density lipoprotein; TyG, triglyceride-glucose

**Fig. 7:**
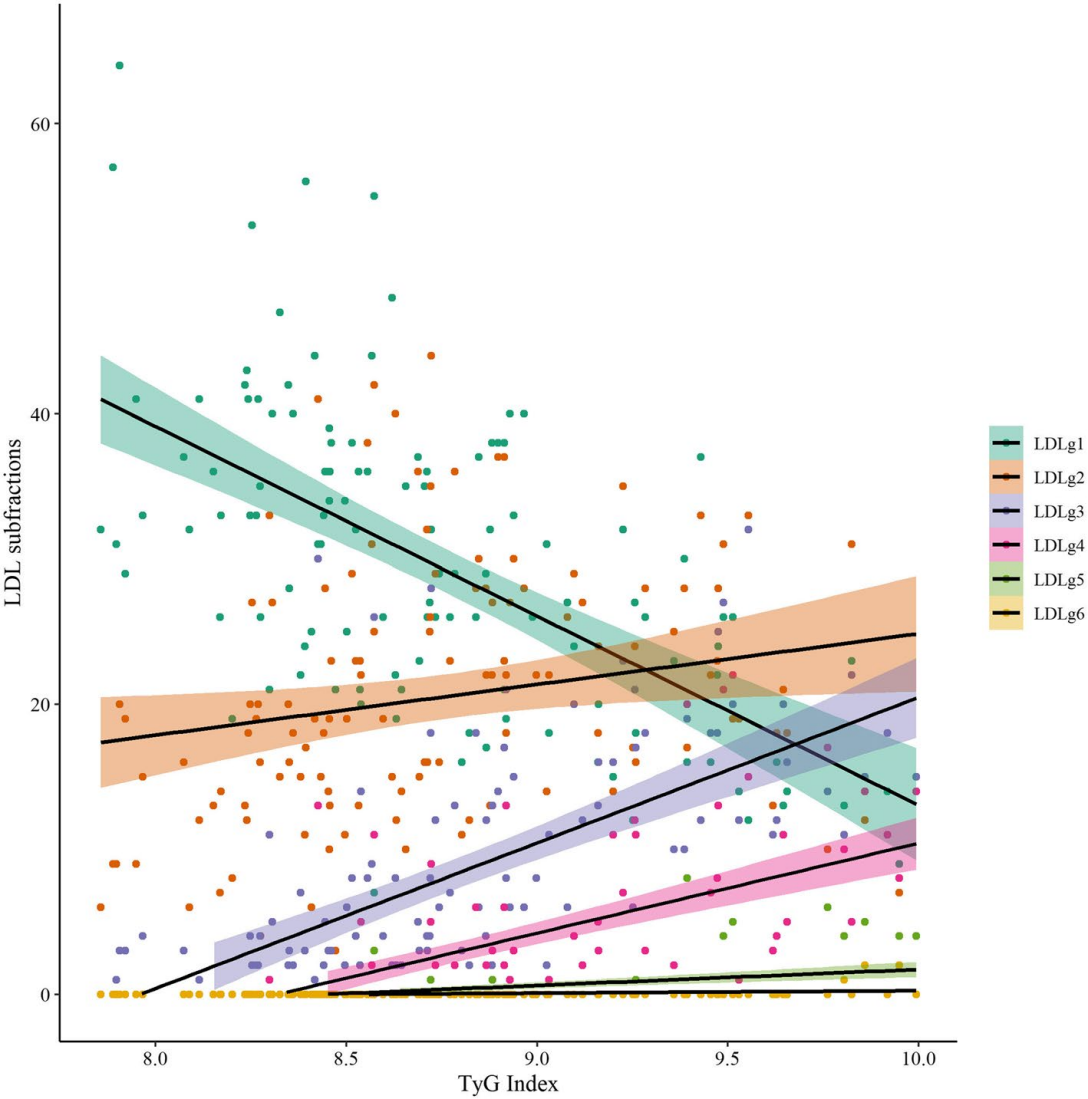
Overall distribution according to the TyG index of LDL subfractions. LDL, low-density lipoprotein; TyG, triglyceride-glucose

A detailed distribution of the LDL subfractions concentrations according to the TyG index obtained through regression analysis was drawn (Fig. 6). Particularly noteworthy were a significant decrease in LDL subclass 1 (R = -0.599, *P* < 0.001) and a notable increase in subclasses 3–4 (R = 0.718, *P* < 0.001, R = 0.683, *P* < 0.001, respectively). Figure 7 illustrates the overall distribution according to the TyG index of LDL subfractions.

## Discussion

In this study, after controlling for relevant confounding variables, an independent link between mean LDL particle size and the TyG index was discovered. And it was also related to other lipid parameters. However, mean LDL particle size was more strongly associated with the TyG index than were other lipid parameters. Additionally, the TyG index cutoff value for predominance of sdLDL particles was 8.72, which is close to the cutoff value for diabetes risk in Koreans.

The evaluation of LDL particles is more accurate in predicting CVD risk than that of LDL-C. LDL particles are varied, and atherogenicity could differ according to their size, composition, and physiochemical properties. LDL particles with a small size (sdLDL-C) are a superior predictor of CVD than LDL-C particles [37]. High sdLDL-C levels not only increase CVD risk in primary prevention subjects but are also considered a risk factor for future events in secondary and/or tertiary prevention in CVD patients [8]. Accordingly, sdLDL-C analysis may benefit a large population given the cost-efficiency and simplicity of the methods. In addition, sdLDL-C levels are strongly affected by environmental factors, which may easily be improved and at little cost through lifestyle modifications [8].

IR can alter lipid metabolism, and sdLDL-C is a characteristic trait of diabetic dyslipidemia [38]. IR increases the triglyceride content of LDL, which promotes LDL lipolysis and enhances the prevalence of more atherogenic sdLDL-C particles [39, 40]. The correlation with other surrogate IR markers and mean LDL particle size has been established in earlier studies; however, the results were inconsistent [19, 41–44]. This may partly be due to the use of different methods for measuring IR and LDL subfractions, which may produce different results. In addition, the results often depended on the triglyceride levels. For example, serum triglyceride concentration and hepatic lipase activity are significant determinants of sdLDL-C in patients with and without mild hypertriglyceridemic diabetes [41, 45]. Moreover, the association of sdLDL-C with insulin sensitivity is strongly modified by triglyceride levels [46].

Therefore, this study assessed the TyG index as a marker of IR obtained by logarithmizing the fasting triglyceride and glucose levels. A previous study has revealed that the TyG index is clinically meaningful in assessing CVD prognosis and risk [22]. Consistently with earlier studies, this study showed that the proportions of people with high FRS increased with higher TyG index quartiles and lower LDL particle size quartiles. The expected results from the trend test were confirmed once again. The mean LDL particle size and the TyG index were associated. Additionally, the TyG index had a greater correlation with mean LDL particle size than had HDL-C, LDL-C, and VLDL-C levels, based on the Steiger’s Z test. These results showed that the TyG index, a convenient and low-cost surrogate marker, could be a robust determinant of the predominance of atherogenic sdLDL particles and can predict atherosclerosis and CVD. Additionally, when compared to other insulin markers, the TyG index’s correlation coefficients with variables and ability to predict LDL particle size were not inferior to those of other markers. Furthermore, the study identified the optimal TyG index cutoff value for sdLDL-C predominance, which is similar to the known diabetes risk cutoff value in Koreans [36]. With the AUC of 0.897, the cutoff value can have a very high predictive power for the subclass pattern B of LDL, which is distinguished by sdLDL-C predominance. To date, no studies have uncovered the mechanism causing the link between sdLDL-C predominance and the TyG index. One possible explanation may be that IR increases the activity of cholesteryl ester transfer protein, which facilitates triglyceride conversion from VLDL-C to LDL-C, leading to the formation of triglyceride-rich LDL-C. This can cause lipolysis by increasing hepatic lipase activity and sdLDL-C [47]. In contrast, IR reduces lipoprotein lipase action, which contributes to VLDL-C clearance, thereby reducing the hepatic uptake of VLDL-C and LDL, causing LDL to remain in the plasma and subsequently increasing sdLDL-C [48]. Recent studies using an animal model of IR have shown that increased expression of microsomal triglyceride transfer protein in the liver, associated with reduced degradation of apolipoprotein B and VLDL-C overproduction, results in an increase in sdLDL-C levels [49–51]. Although the detailed mechanism is not fully understood, this research shows that a decline in LDL subclass 1 and an elevation in LDL subclasses 3–4, in particular, play a crucial part in the increase in sdLDL-C predominance.

### Strengths and limitations

This research indicates various strengths. First, this is the only research to figure out the connection between the TyG index and the predominance of sdLDL-C. Second, this study revealed that the TyG index was not inferior when compared to other IR markers including triglyceride/HDL-C levels. Also, the TyG index provides an extensive assessment of both lipid and glucose metabolism than does triglyceride/HDL-C, a well-known IR marker for assessing the presence of sdLDL-C [52–54]. Third, the benefit of the TyG index is being an IR that is economical and relies on preexisting data. It is inefficient to test LDL subfractions to assess sdLDL-C clinically, and the TyG index has the advantage that sdLDL-C predominance can be easily estimated through preexisting data in primary care settings. Fourth, it is encouraging that the presented cutoff value of the TyG index could predict the LDL subclass pattern B predominance. The LDL subclass pattern B is known to be the most important cause of atherosclerosis and CVD, so using the TyG index as a screening tool could be beneficial.

The limitations of the research are as follows. First, the research could not assess causality or temporality due to its observational cross-sectional design, and exercise and food habits, for example, were not ruled out as potential residual confounding factors. Although these factors are important in metabolism-related studies, their evaluation was not available due to limitations in the research method. Second, selection bias may have occurred and affected the outcomes because the research sample consisted of data gathered from one healthcare facility, which could not be considered generic population representative. Although participants in this research exceeded the conservatively determined sample size, the prevalence was not accurately known, and the participants were predominantly female, potentially reducing the applicability of our results to the general population. Finally, as LDL particle size could not be calculated directly, this research used the Lipoprint system for indirect measurement, which was simple and clinically accessible. Therefore, the concentrations of sdLDL-C and lbLDL-C obtained with the LDL subfractions were corrected to match the LDL-C concentrations obtained with the chemical analyzer. Despite these limitations, this is the only study that examined the association between the TyG index and sdLDL-C predominance independently of the triglyceride levels.

## Conclusion

This study found that mean LDL particle size was more strongly correlated with the TyG index than were other lipid parameters. After correcting for related confounding variables, the research discovered for the first time that the mean LDL particle size was independently correlated with the TyG index. Moreover, the TyG index, a simple and fast IR biomarker, was associated with predominance of atherogenic sdLDL particles. In the clinical setting, the TyG index may be used to presume the predominance of sdLDL-C particles without requiring expensive tests, such as LDL subfractions analysis, in patients with or without dyslipidemia to identify CVD risk. The TyG index could also be used to evaluate disease progression and outcome in existing CVD patients. Longitudinal studies with larger datasets are required to confirm the results. Future studies should clarify the clinical benefit of reducing the TyG index to evaluate the sdLDL subfraction and predicting CVD risk.

## Supplementary Information

**Additional file 1. **Relationship between the triglyceride-glucose (TyG) index and clinical metabolic variables. Comparison of correlation coefficients for clinical metabolic variables and other insulin markers. Multiple linear regression analysis to determine relationships between the other insulin markers and clinical metabolic variables. 

**Additional file 2. **Relationship between abdominal visceral and abdominal subcutaneous fat areas measured using CT scan and LDL particle size.

## Data Availability

The datasets used and/or analyzed during the current study are available from the corresponding author on reasonable request.

## Abbreviations

ALT: Alanine transaminase
AST: Aspartate transaminase
AUC: Area under the curve
BMI: Body mass index
CI: Confidence interval
CT: Computed tomography
CVD: Cardiovascular disease
FRS: Framingham risk score
GGT: Gamma-glutamyl transferase
HDL-C: High-density lipoprotein cholesterol
HOMA-IR: Homeostasis model assessment estimated insulin resistance
hsCRP: High-sensitivity C-reactive protein
IR: Insulin resistance
lbLDL: Large, buoyant low-density lipoprotein cholesterol
LDL: Low-density lipoprotein
LDL-C: Low-density lipoprotein cholesterol
Rf: retention factor
ROC: Receiver operating characteristic
sdLDL-C: Small dense low-density lipoprotein cholesterol
SE: Standard error
TyG: Triglyceride-glucose
VLDL-C: Very low-density lipoprotein cholesterol
