# Supplementary material for: Association between triglyceride-glucose index and low-density lipoprotein particle size in korean obese adults
Source: Lipids Health Dis. 2023 Jul 4;22:94. doi: 10.1186/s12944-023-01857-5 (PMC10318677; doi:10.1186/s12944-023-01857-5)
Supplement: Supplementary file 1 — Additional file 1. Relationship between the triglyceride-glucose (TyG) index and clinical metabolic variables. Comparison of correlation coefficients for clinical metabolic variables and other insulin markers. Multiple linear regression analysis to determine relationships between the other insulin markers and clinical metabolic variables. [file 12944_2023_1857_MOESM1_ESM.docx]

Additional file 1-1. Relationship between the triglyceride-glucose (TyG) index and clinical metabolic variables

| Variable | Correlation Coefficient | *P*-value |
| --- | --- | --- |
| Age (year) | -0.004 | 0.964 |
| Body mass index (kg/m²) | 0.153 | 0.095 |
| Waist circumference (cm) | 0.262 | 0.004 |
| Systolic blood pressure (mmHg) | 0.136 | 0.136 |
| Diastolic blood pressure (mmHg) | 0.144 | 0.113 |
| Heart rate (beats/min) | 0.059 | 0.516 |
| AST (IU/L) | 0.33 | <0.001 |
| ALT (IU/L) | 0.335 | <0.001 |
| GGT (IU/L) | 0.428 | <0.001 |
| hsCRP (mg/L) | 0.066 | 0.468 |
| Uric acid (mg/dL) | 0.36 | <0.001 |
| Insulin (μU/mL) | 0.401 | <0.001 |
| HOMA-IR | 0.412 | <0.001 |
| Abdominal visceral fat area (cm^2^) | 0.285 | 0.002 |
| Abdominal subcutaneous fat area (cm^2^) | 0.103 | 0.281 |
| Framingham risk score | 0.315 | <0.001 |
| Abbreviations: AST, aspartate aminotransferase; ALT, alanine aminotransferase; GGT, gamma-glutamyl transferase; hsCRP, high-sensitivity C-reactive protein; HOMA-IR, homeostasis model assessment-estimated insulin resistance. | | |

Additional file 1-2. Comparison between correlation coefficients for the other insulin markers.

| Insulin marker  Variable | HOMA-IR | | Insulin | | Triglyceride | | Glucose | | Triglyceride/HDL-C | |
| --- | --- | --- | --- | --- | --- | --- | --- | --- | --- | --- |
|  | Correlation  Coefficient | *P*-value | Correlation  Coefficient | *P*-value | Correlation  Coefficient | *P*-value | Correlation  Coefficient | *P*-value | Correlation  Coefficient | *P*-value |
| Age (year) | -0.196 | 0.028 | -0.209 | 0.019 | -0.014 | 0.873 | 0.058 | 0.518 | -0.068 | 0.446 |
| Body mass index (kg/m²) | 0.457 | <0.001 | 0.47 | <0.001 | 0.141 | 0.124 | 0.149 | 0.104 | 0.209 | 0.022 |
| Waist circumference (cm) | 0.45 | <0.001 | 0.457 | <0.001 | 0.252 | 0.006 | 0.197 | 0.034 | 0.315 | <0.001 |
| Systolic blood pressure (mmHg) | 0.189 | 0.039 | 0.186 | 0.042 | 0.129 | 0.158 | 0.154 | 0.09 | 0.094 | 0.301 |
| Diastolic blood pressure (mmHg) | 0.193 | 0.035 | 0.182 | 0.047 | 0.136 | 0.135 | 0.219 | 0.015 | 0.096 | 0.295 |
| Heart rate (beats/min) | 0.287 | 0.001 | 0.285 | 0.002 | 0.033 | 0.717 | 0.171 | 0.06 | 0.039 | 0.672 |
| AST (IU/L) | 0.249 | 0.005 | 0.263 | 0.003 | 0.348 | <0.001 | -0.002 | 0.986 | 0.391 | <0.001 |
| ALT (IU/L) | 0.388 | <0.001 | 0.386 | <0.001 | 0.32 | <0.001 | 0.159 | 0.073 | 0.396 | <0.001 |
| GGT (IU/L) | 0.377 | <0.001 | 0.359 | <0.001 | 0.396 | <0.001 | 0.267 | 0.003 | 0.41 | <0.001 |
| hsCRP (mg/L) | 0.366 | <0.001 | 0.377 | <0.001 | 0.067 | 0.459 | 0.059 | 0.517 | 0.116 | 0.203 |
| Uric acid (mg/dL) | 0.339 | <0.001 | 0.351 | <0.001 | 0.372 | <0.001 | 0.035 | 0.695 | 0.416 | <0.001 |
| Insulin (μU/mL) | 0.991 | <0.001 | 1 | <0.001 | 0.364 | <0.001 | 0.361 | <0.001 | 0.436 | <0.001 |
| HOMA-IR | 1 | <0.001 | 0.991 | <0.001 | 0.357 | <0.001 | 0.462 | <0.001 | 0.426 | <0.001 |
| Abdominal visceral fat area (cm^2^) | 0.321 | <0.001 | 0.338 | <0.001 | 0.297 | 0.002 | 0.047 | 0.628 | 0.258 | 0.006 |
| Abdominal subcutaneous fat area (cm^2^) | 0.317 | <0.001 | 0.335 | <0.001 | 0.117 | 0.22 | 0.047 | 0.627 | 0.125 | 0.191 |
| Framingham risk score | 0.06 | 0.52 | 0.038 | 0.68 | 0.297 | <0.001 | 0.233 | 0.01 | 0.296 | <0.001 |

Abbreviations: AST, aspartate aminotransferase; ALT, alanine aminotransferase; GGT, gamma-glutamyl transferase; hsCRP, high-sensitivity C-reactive protein; HOMA-IR, homeostasis model assessment-estimated insulin resistance; HDL-C, high-density lipoprotein cholesterol.

Additional file 1-3. Multiple linear regression analysis to determine relationships between the other insulin markers and clinical metabolic variables

|  | HOMA-IR | | | Insulin | | | TG | | | Glucose | | | TG/HDL-C | | |
| --- | --- | --- | --- | --- | --- | --- | --- | --- | --- | --- | --- | --- | --- | --- | --- |
|  | β coefficient | 95% CI | p-value | β coefficient | 95% CI | *P* -value | β coefficient | 95% CI | *P* -value | β coefficient | 95% CI | *P*-value | β coefficient | 95% CI | *P* -value |
| Age (year) | 0.041 | (-0.121, 0.202) | 0.618 | -0.068 | (-0.241, 0.105) | 0.436 | -0.052 | (-0.835, 0.731) | 0.895 | 0.097 | (-0.077, 0.271) | 0.27 | -0.01 | (-0.029, 0.009) | 0.301 |
| Sex |  |  |  |  |  |  |  |  |  |  |  |  |  |  |  |
| Male | Ref |  |  |  |  |  |  |  |  |  |  |  |  |  |  |
| Female | -0.607 | (-4.565, 3.351) | 0.762 | 1.052 | (-3.185, 5.289) | 0.623 | 12.163 | (-7.012, 31.338) | 0.211 | -0.142 | (-4.399, 4.116) | 0.947 | -0.031 | (-0.505, 0.442) | 0.895 |
| Body mass index (kg/m²) | -0.144 | (-0.514, 0.226) | 0.443 | 0.651 | (0.275, 1.027) | <0.001 | -1.662 | (-3.454, 0.131) | 0.069 | -0.174 | (-0.572, 0.224) | 0.389 | -0.035 | (-0.079, 0.009) | 0.117 |
| Alcohol consumption |  |  |  |  |  |  |  |  |  |  |  |  |  |  |  |
| Yes | -0.629 | (-4.506, 3.249) | 0.748 | 2.101 | (-2.035, 6.237) | 0.316 | 9.38 | (-9.407, 28.166) | 0.324 | -2.061 | (-6.232, 2.11) | 0.329 | -0.044 | (-0.508, 0.419) | 0.85 |
| No | Ref |  |  |  |  |  |  |  |  |  |  |  |  |  |  |
| Smoking history |  |  |  |  |  |  |  |  |  |  |  |  |  |  |  |
| Yes | 3.421 | (-2.075, 8.916) | 0.22 | 2.424 | (-3.447, 8.295) | 0.415 | 10.976 | (-15.649, 37.601) | 0.415 | 5.339 | (-0.573, 11.25) | 0.076 | 0.53 | (-0.127, 1.187) | 0.113 |
| No | Ref |  |  |  |  |  |  |  |  |  |  |  |  |  |  |
| Systolic blood pressure (mmHg) | -0.11 | (-0.305, 0.085) | 0.267 | -0.043 | (-0.252, 0.166) | 0.685 | -0.076 | (-1.021, 0.868) | 0.873 | -0.116 | (-0.326, 0.094) | 0.275 | -0.003 | (-0.027, 0.02) | 0.769 |
| Diastolic blood pressure (mmHg) | 0.299 | (0.037, 0.561) | 0.026 | 0.042 | (-0.239, 0.323) | 0.766 | 0.362 | (-0.909, 1.632) | 0.573 | 0.322 | (0.04, 0.605) | 0.025 | 0.007 | (-0.024, 0.038) | 0.652 |
| LDL-C (mg/dL) | 0.022 | (-0.034, 0.077) | 0.441 | -0.056 | (-0.114, 0.003) | 0.061 | 0.166 | (-0.102, 0.435) | 0.223 | 0.049 | (-0.01, 0.109) | 0.104 | 0.002 | (-0.005, 0.008) | 0.577 |
| VLDL-C (mg/dL) | 0.587 | (0.231, 0.944) | 0.001 | 0.083 | (-0.299, 0.464) | 0.668 | 4.94 | (3.213, 6.666) | <0.001 | 0.67 | (0.287, 1.054) | <0.001 | 0.141 | (0.099, 0.184) | <0.001 |
| Mean LDL particle size (Å) | 0.108 | (-0.16, 0.377) | 0.426 | -0.412 | (-0.688, -0.136) | 0.004 | -6.743 | (-8.044, -5.442) | <0.001 | 0.312 | (0.023, 0.601) | 0.035 | -0.169 | (-0.201, -0.137) | <0.001 |
| Uric acid (mg/dL) | -2.043 | (-3.462, -0.625) | 0.005 | 0.985 | (-0.524, 2.493) | 0.198 | 2.637 | (-4.237, 9.51) | 0.448 | -1.312 | (-2.838, 0.214) | 0.091 | 0.079 | (-0.09, 0.249) | 0.355 |
| Insulin (μU/mL) | 4.652 | (4.47, 4.834) | <0.001 |  |  |  | 1.676 | (0.792, 2.559) | <0.001 | 0.352 | (0.156, 0.548) | <0.001 | 0.037 | (0.015, 0.059) | 0.001 |

Abbreviations: CI, confidence interval, LDL, low-density lipoprotein; LDL-C, low-density lipoprotein cholesterol; TG, triglyceride; VLDL-C, very low-density lipoprotein cholesterol;
