# Supplementary figures and images for: Association between triglyceride-glucose index and low-density lipoprotein particle size in korean obese adults
Source: Lipids Health Dis. 2023 Jul 4;22:94. doi: 10.1186/s12944-023-01857-5 (PMC10318677; doi:10.1186/s12944-023-01857-5)

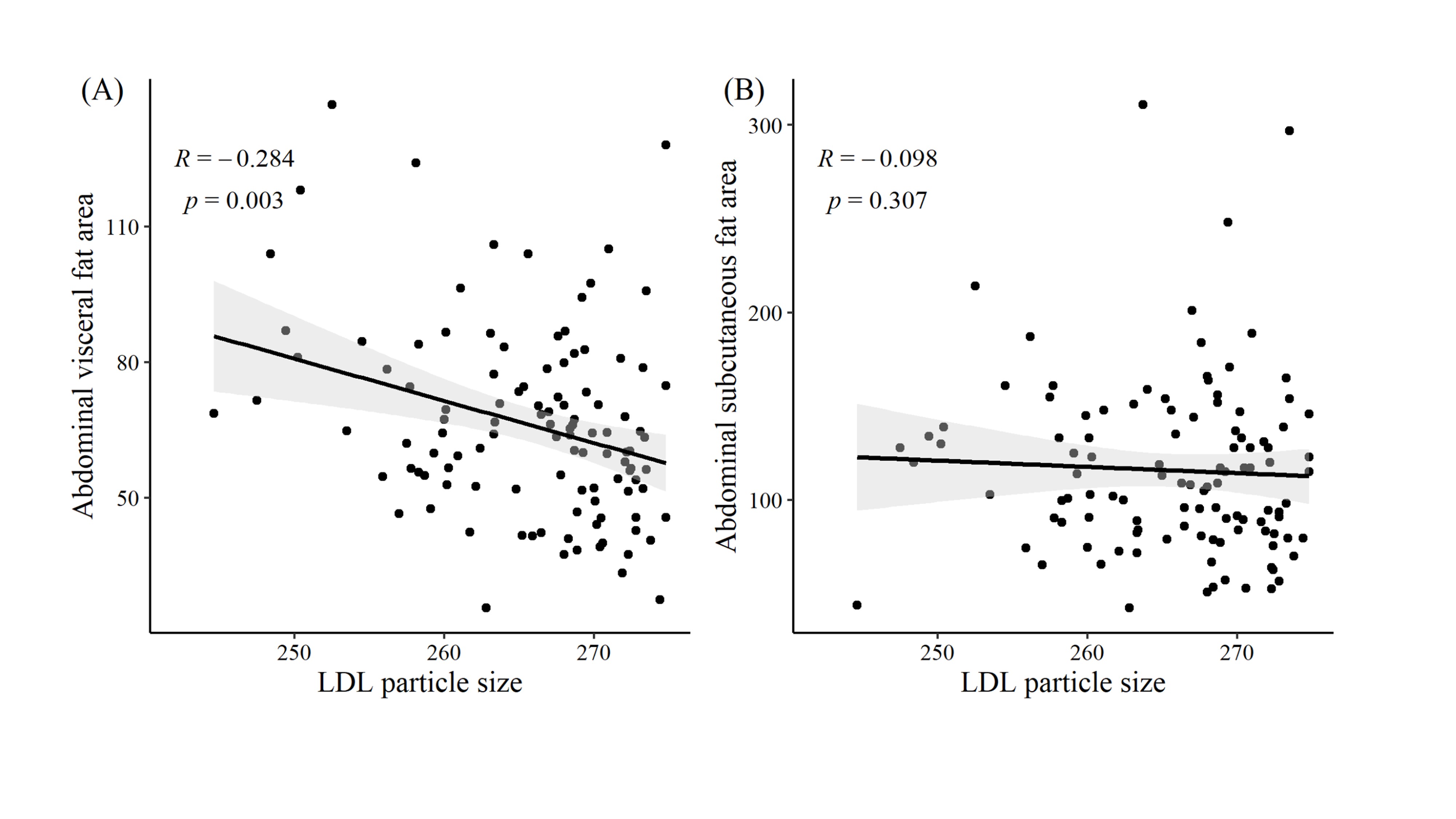

Supplement: Supplementary file 2 — Additional file 2. Relationship between abdominal visceral and abdominal subcutaneous fat areas measured using CT scan and LDL particle size. [file 12944_2023_1857_MOESM2_ESM.jpg]
